# HTLV-1 Tax1 represses the proapoptotic protein Bim, which is crucial for T-cell transformation

**DOI:** 10.1186/1742-4690-8-S1-A152

**Published:** 2011-06-06

**Authors:** Masaya Higuchi, Masahiro Fujii

**Affiliations:** 1 Division of Virology, Niigata University Graduate School of Medical and Dental Sciences, Niigata, 951-8510, Japan

## 

Human T cell leukemia virus type 1 (HTLV-1) is an etiological agent of adult T-cell leukemia (ATL) and its viral oncoprotein Tax1 plays critical roles in T cell transformation and ATL development. Tax1 converts a T-cell line CTLL-2 from IL-2 dependent growth into IL-2-independent one, and the conversion requires inhibition of apoptosis. In this study, we investigated the involvement of Bim, an apoptosis inducer after cytokine deprivation, in Tax1-induced transformation. Bim expression is strongly induced in CTLL-2 cells after IL-2 starvation, whereas such induction is markedly reduced in CTLL-2 cells transformed by Tax1. A Tax1 mutant defective of NF-kB2 activation transformed CTLL-2 less efficiently than wild type Tax, but this low transforming activity was rescued by knockdown of Bim in CTLL-2, suggesting that activation of noncanonical NF-kB2 pathway is involved in Tax1-mediated Bim repression. Furthermore, the expression of Bim was lower in HTLV-1 transformed and ATL cell lines than HTLV-1-negative T-cell lines. These results suggest that the Bim repression by Tax1 is crucial for the survival of HTLV-1 infected cells and may play a role in ATL development.

